# Association of Red Cell Distribution Width-to-Platelet Ratio and Mortality in Patients with Sepsis

**DOI:** 10.1155/2022/4915887

**Published:** 2022-09-27

**Authors:** Jie Liu, Xueying Huang, Suru Yue, Jia Wang, Enlin Ye, Jiasheng Huang, Yumei Zhao, Dongdong Niu, Xuefei Hou, Jiayuan Wu

**Affiliations:** ^1^Clinical Research Service Center, Affiliated Hospital of Guangdong Medical University, Zhanjiang, 524001 Guangdong, China; ^2^Collaborative Innovation Engineering Technology Research Center of Clinical Medical Big Data Cloud Service in Medical Consortium of West Guangdong Province, Affiliated Hospital of Guangdong Medical University, Zhanjiang, 524001 Guangdong, China

## Abstract

**Background:**

As a novel inflammatory index, the ratio of red cell distribution width (RDW) to platelet count (RPR) may have prognostic value in some critical illnesses. However, studies on the prognostic influence of RPR in patients with sepsis are few. This study is aimed at investigating the association between RPR levels and 28-day mortality in patients with sepsis.

**Methods:**

Data of patients with sepsis were obtained from the Medical Information Mart for Intensive Care III database. The best cut-off value was calculated by establishing the receiver operating characteristic curve (ROC), and the predictive ability of different indicators was compared through the area under the curve (AUC). The association between RPR levels and 28-day mortality was assessed using the Cox proportional hazards model. Restrictive cubic spline analysis was applied to the multivariable Cox model to investigate the nonlinear relationship between RPR and 28-day mortality.

**Results:**

A total of 3367 patients with sepsis were included in the study. A nonlinear relationship was observed between RPR and 28-day mortality, showing a trend of a first rapid increase and a gradual increase. For the prediction of mortality, the best cut-off value for RPR was 0.109, with an AUC of 0.728 (95% confidence interval [CI]: 0.709–0.747). The predictive capability of RPR was superior to those of RDW, platelet, SOFA score, and SAPS II score. After adjusting for various confounding factors, high RPR was significantly associated with increased mortality with adjusted hazard ratios of 1.210 (95% CI: 1.045–1.400) for categorical variables and 2.826 (95% CI: 2.025–3.944) for continuous variables.

**Conclusion:**

Elevated RPR level is significantly correlated with a high risk of 28-day mortality in patients with sepsis and can be a new predictor of patient prognosis.

## 1. Introduction

Sepsis affects disease progression in critically ill patients and is one of the major causes of death. The incidence rate of sepsis is as high as 535/100,000 with a mortality rate up to 25%–30% among hospitalized patients [1]. Despite significant developments in the pathophysiology and therapeutic strategies of sepsis, the mortality caused by sepsis is still high [2]. The complex systemic inflammation and anti-inflammatory response play a key part in the pathophysiological process of sepsis [3]. Many studies showed that the delay of diagnosis and treatment is significantly related to the high mortality of sepsis [4]. Identifying high-risk patients with poor prognosis at the early stage and carrying out timely and effective intervention are critical for improving survival outcome. Therefore, convenient and reliable predictors should be explored to improve the prognostic management of patients with sepsis.

Therefore, evaluating the severity degree and prognostic status of patients with sepsis can be performed using some single- and multiple-parameter biomarkers (including lactic acid, chloride, neutrophil-to-albumin ratio, platelet-to-lymphocyte ratio, and neutrophil-to-lymphocyte ratio) [3, 5–8] and scoring systems (including Acute Physiology Score III [APS III], sequential organ failure assessment [SOFA], and Simplified Acute Physiology Score II [SAPS II]). Unfortunately, single-parameter biomarkers can be affected by many factors, such as malignancy, immune factors, and drug types, leading to an unsatisfactory predictive performance [9]. Moreover, the application value of most multiparameter biomarkers is limited because of low sensitivity or specificity.

The red cell distribution width (RDW) is a parameter reflecting the variation and dispersion degree of peripheral blood red blood cell volume. The elevation of RDW has important clinical significance in many fields. Recent research [9–11] indicated that RDW is strongly associated with prognosis in acute kidney injury (AKI), severe burn, and acute respiratory distress syndrome. Platelets are small circulating nuclear cells with biological activity that break off from the cytoplasm of mature megakaryocytes in bone marrow. Platelets play a significant part in the process of hemostasis. A previous research showed that septic patients with low platelet counts at admission are more severely ill and have higher risk of death than patients with normal platelet counts [12]. Thrombocytopenia, which affects unfavorable prognosis in patients with sepsis, is an independent hazard factor for sepsis [13]. Given that changes in RDW and platelets are significant parts of blood pathophysiology during sepsis, these changes are usually complementary rather than isolated. Recently, a new risk parameter incorporating RDW and platelet counts, i.e., RDW-to-platelet ratio (RPR), is already applied to predict prognosis in patients with severe burns [10], breast cancer [14], acute pancreatitis [15], and acute kidney injury [16]. Nevertheless, research on the effect of RPR in prediction of prognosis in patients with sepsis is few. This research aims to evaluate the relationship of RPR and mortality in patients with sepsis.

## 2. Methods

### 2.1. Data Source

This study was carried out using a free and open clinical database, namely, the Medical Information Mart for Intensive Care III (MIMIC-III). This database collected the information of more than 50,000 patients in various intensive care units (ICUs) at Beth Israel Deaconess Medical Center (BIDMC; Boston, USA) from 2001 to 2012. This information included general demographic information, vital signs, laboratory parameters, drugs, nursing records, and other clinically relevant variables. Institutional review boards of the Massachusetts Institute of Technology and BIDMC authorized the study. To gain the right of access to the database, we took an exam called Protection Human Research Participants and received a numbered certificate (No. 9986104). All personal information is already hidden to protect patient privacy.

### 2.2. Population Selection Criteria

Adult patients who met the criteria for sepsis in accordance with the International Classification of Diseases-9 code (“99591”, “99592”, and “78552”) were initially screened. Inclusion criteria were as follows: (1) age ≥ 18 years old and (2) admission to ICU for more than two days. Patients were excluded from this study in accordance with the exclusion criteria: (1) lack of data on RDW and platelets during ICU stay, (2) age ≥ 89 years, (3) more than 5% of personal data missing, and (4) received treatment that may affect RDW and PLT (such as platelet raising drugs) or combined with underlying diseases that may affect RDW and PLT (such as blood diseases). For patients with multiple ICU admissions, only the first-time ICU admission was selected for this research.

### 2.3. Data Extraction

Structured Query Language (SQL) and the PostgreSQL tool were used to extract data from the MIMIC-III. Extracted data included demographic information, life signs, complications, laboratory indicators, clinical scoring system, and therapeutic management. Demographic data included age, gender, ethnicity, marital status, and body mass index. Complications included congestive heart failure (CHF), hypertension, and diabetes. Laboratory indicators included albumin, bicarbonate, anion gap, creatinine, bilirubin, chloride, glucose, hematocrit, hemoglobin, sodium, potassium, blood urea nitrogen (BUN), white blood cell, lactate, prothrombin time (PT), international normalized ratio (INR), lymphocytes, neutrophils, RDW, and PLT levels. SOFA and SAPS II scores were determined for every patient. Vital signs included body temperature and SpO_2_. Therapeutic management included renal replacement therapy (RRT), mechanical ventilation, and vasopressin use. All data were extracted within 24 hours after ICU admission. The 28-day mortality was regarded as the survival endpoint.

### 2.4. Study Sample Size

Given that this study was a hypothesis-driven exploratory study based on a publicly available database, no attempt was made to estimate the necessary sample size for the study. Instead, all eligible patients in the MIMIC-III database were included to achieve the maximum statistical power.

### 2.5. Statistical Analysis

Continuous variables were presented as median with interquartile range (IQR), and categorical variables were presented as number of cases or percentage (%). The chi-square test was used to compare categorical variables, and the Wilcoxon rank-sum test was used to compare continuous variables. We used the receiver operating characteristic curve (ROC) to determine the area under curve (AUC) and assess the distinction among various variables on mortality. ROC was also applied to calculate the best cut-off value of RPR by showing the conversion between specificity and sensitivity. AUC was compared using the *Z*-test to show the distinction in prediction performance among various indicators. The association between RPR levels and 28-day mortality was assessed using the Cox proportional hazards model. The results of the Cox regression were expressed as hazard ratio (HR) with 95% confidence interval (CI). When RPR was a continuous variable, a restricted cubic spline analysis of the Cox model was performed on all covariates, and the shape showed the association between RPR and mortality.

The R software version 4.1.1 was applied for statistical analysis. A two-sided *P* < 0.05 indicated statistical significance.

## 3. Results

### 3.1. Baseline Characteristics

Patient baseline characteristics are summarized in Table 1. We extracted 3367 patients with sepsis in the study. These cases included 1917 (56.9%) males and 2449 (72.7%) whites.

In accordance with their survival, patients were separated into two groups. A total of 960 and 2407 patients belonged to the nonsurvivor and survivor groups, respectively. As shown in Table 1, age, marital status, ethnicity, body temperature, RRT, mechanical ventilation, vasopressor use, albumin, bicarbonate, SpO_2_, anion gap, creatinine, lactate, bilirubin, chloride, lymphocytes, hematocrit, hemoglobin, neutrophils, sodium, potassium, BUN, PT, INR, CHF, and RPR of survivors were lower than those of the nonsurvivors (*P* < 0.05).

### 3.2. Predictive Value of RPR for 28-Day Mortality

**Figure 1:**
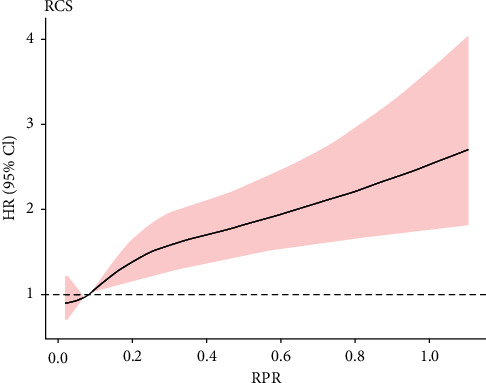
The relationship between RPR levels and 28-d mortality in patients with sepsis was plotted using multivariable adjusted restricted cubic splines. There was a nonlinear relationship between RPR and 28-d mortality, showing a trend of rapid first and then gradually increasing, that is, the higher the RPR level, the higher the mortality risk. The range area represents a 95% confidence interval. HR: hazard ratio; CI: confidence interval; RPR: red cell distribution width to platelet ratio.

RPR has a nonlinear relationship with mortality with a trend to rapidly and then gradually, indicating that high RPR levels resulted in high risk of death (Figure 1).

**Figure 2:**
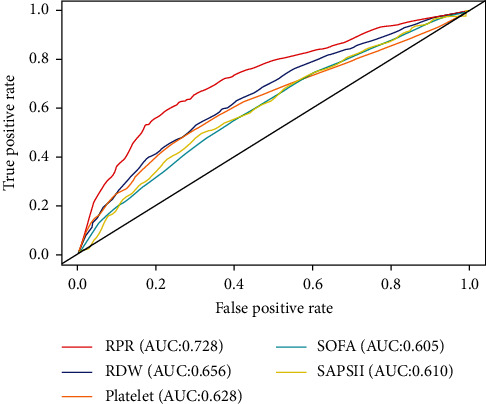
Receiver operating characteristics curves of RPR, RDW, platelet, SOFA, and SAPS II score for predicting 28-d mortality in patients with sepsis. The predictive ability of PRR for 28-d mortality outperformed other indices, including RDW, platelet, SOFA score, and SAPS II score by comparing the area under the curve. RPR: red cell distribution width to platelet ratio; RDW: red cell distribution width; SOFA: sequential organ failure assessment; SAPS II: simplified acute physiology score II.

The ROC analysis was used to evaluate the prediction capability of RPR, RDW, platelet, SOFA, and SAPS II scores for 28-day mortality (Figure 2). The best cut-off value for RPR to predict mortality was 0.109 with specificity of 0.777 and sensitivity of 0.588 (Table 2). The AUC of RPR was 0.728 (95% CI: 0.709–0.747), which was clinically higher than those of RDW (AUC = 0.656, 95% CI: 0.636–0.677, *Z* = 5.871, *P* < 0.001), platelet (AUC = 0.628, 95% CI: 0.606–0.649, *Z* = 12.357, *P* < 0.001), SOFA score (AUC = 0.605, 95% CI: 0.584–0.626, *Z* = 7.876, *P* < 0.001), and SAPS II score (AUC = 0.610, 95% CI: 0.589–0.631, *Z* = 8.601, *P* < 0.001 (Table 2)).

### 3.3. Association between RPR and Mortality

**Figure 3:**
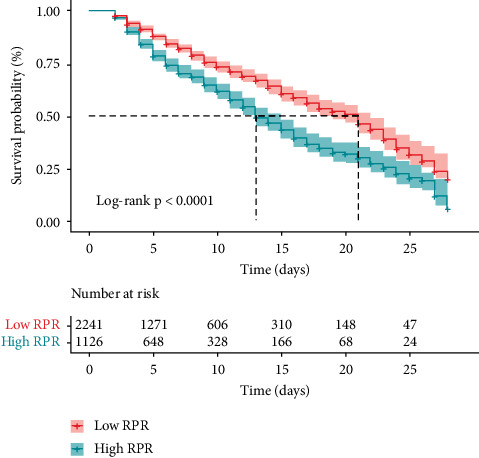
Kaplan-Meier analysis of 28-d mortality by the RPR levels in patients with sepsis. The 28-d mortality rate in the high RPR group was significantly higher than that in the low RPR group (log-rank test, *P* < 0.001). RPR: red cell distribution width to platelet ratio.

Figure 3 shows the results of the Kaplan–Meier method concerning the 28-day mortality analysis by RPR levels. The 28-day mortality in the low-RPR group was evidently lower than that in the high-RPR group (log rank test, *P* < 0.001).

To explore the association between RPR and mortality, we conducted the Cox regression analysis. A rough model of univariate analysis showed that a high level of RPR was notably correlated with a high risk of mortality with HR of 1.607 (95% CI: 1.415–1.825) for categorical variables and 2.897 (95% CI: 2.216–3.788) for continuous variables (Table 3, crude model). Multivariable analysis showed that septic patients with high RPR levels had a high risk of 28-day mortality during hospitalization even when demographic characteristics, complications, laboratory test indicators, clinical treatment, and scoring system were adjusted (Table 3, models 1–5). In the fully adjusted model, when RPR was analyzed as categorical and continuous variables, the HRs of the RPR mortality risk were 1.210 (95% CI: 1.045–1.400) and 2.826 (95% CI: 2.025–3.944), respectively (Table 3, model 5).

## 4. Discussion

We have found a significant relationship between elevated RPR levels and increasing 28-day mortality in 3367 patients with sepsis. A nonlinear association is observed between RPR and mortality risk, showing a trend of rapidly increase and then gradually. Thus, RPR can be a potential predictive factor of 28-day mortality in patients with sepsis. The predictive effect of RPR for mortality is better than those of RDW, platelet, SOFA score, and SAPS II score.

Sepsis is a serious clinical disease and involves the systemic response to infection and causes irreversible and serious damage to cells and tissues. Sepsis usually occurs after an infection that cannot be controlled and cleared by the host. The increase in RDW may be related to a variety of potential metabolic abnormalities, such as inflammation, oxidative stress, telomere shortening, malnutrition, dyslipidemia, hypertension, erythrocyte fragmentation, and abnormality of erythropoietin function [17]. Salvagno et al. observed a significant relationship between RDW and inflammatory biological markers [18]. In the inflammatory response, proinflammatory cytokines affect the survival of red blood cells in the bloodstream, destroy cytomembrane, and inhibit maturation, which can cause larger and newer immature red blood cells to enter the peripheral circulation and increase the RDW. Moreover, high oxidative stress status may decrease the survival rate of erythrocytes, and the shortening of erythrocyte life may stimulate the release of immature erythrocytes, which are bigger than mature erythrocytes from bone marrow to circulation. This phenomenon results in increased RDW [19]. RDW has been shown to be an independent prognostic indicator of sepsis in many previous studies [20–22]. The increase in RDW at admission may be related to short- and long-term adverse consequences in patients with sepsis.

Immune disorders (particularly cell-mediated immunity), including pro- or anti-inflammatory response, are present in most patients with sepsis [23]. Lately, research showed that platelets play a crucial part in immune regulation and the progression of inflammation [24, 25] through the induction of inflammatory cytokine release [26] and the interplay of many different types of germs and immune cells, including T-cells, neutrophils, natural killer cells, and macrophagocyte cell, helping to initiate or deteriorate the inflammatory process [27]. Tissue factor, another important factor of inflammation, induces coagulation dysfunction and leads to decreased platelet count [19]. Low platelet counts may be related to an unfavorable prognosis in the case of sepsis [3]. One major survey of 931 patients with sepsis determined the relationship between thrombocytopenia and prognosis [12]. Thrombocytopenia, one of the most prevalent diseases in sepsis, is a hemorrhagic disease associated with platelet consumption and high mortality in patients. Patients with low platelet counts at ICU admission have more serious conditions, higher APACHE IV and SOFA scores, more shock and organ failure, and higher risk of death. In addition, patients with low platelet counts have increased risk of mortality within one year of ICU admission.

A single index is easily affected by other factors. As a composite index, RPR can timely respond to the dynamic changes in RDW and platelets and can neutralize the influence of other factors. In clinical application, RPR may serve as an important reference index for the prognosis of patients with sepsis and improve the accuracy of the prognostic prediction of sepsis. RPR has certain predictive value for inflammatory diseases, which can be easily calculated [28, 29]. Wu et al. [16] found that the RPR level of newborns with confirmed or suspected early-onset sepsis is significantly higher than that of healthy newborns. Compared with other biomarkers used to diagnose neonatal sepsis, RPR has higher specificity and positive predictive value. RPR is one of the newest markers of inflammatory reaction and has been widely used in the prognostic prediction in several diseases. This index is easy to assess, making it a potentially valuable predictor [30]. By combining the prognostic advantages of RDW and PLT, RPR is considered a new indicator reflecting the severity of inflammation [10]. RPR has shown good clinical diagnostic value and accuracy in predicting significant fibrosis, advanced fibrosis, and liver cirrhosis in patients with chronic liver disease [31]. Arora et al. [32] found that at an early stage, RPR is an economical and reliable marker for prognosticating the severity in patients with acute pancreatitis with high diagnostic accuracy.

Thus far, some scoring systems, such as SOFA and SAPS II scores, have been applied extensively in medical practice to predict the prognosis of critically ill patients. Nevertheless, due to poor prediction performance, the application of these scoring systems is controversial. Simpson et al. [33] suggested that the qSOFA or SOFA score may fail to identify early and intervene in potentially life-threatening infections, resulting in delayed diagnosis and intervention of patients. Judgement standard for organ dysfunction (such as SOFA) needs clinical data and laboratory parameters, which may be rough to gain in time [34]. SOFA and SAPS II scores have many indicators, and the operation process is cumbersome. These scores cannot be quickly obtained in some grassroots hospitals to evaluate the patient's condition. Therefore, limitations in daily use remain [35]. By contrast, RPR requires only the two most common hematological parameters (RDW and PLT). RPR is simple, easy to obtain, rapidly measured, and noninvasive with relatively high accuracy. Thus, RPR can be used as a convenient index in the prognostic prediction.

Some limitations of this study should be considered. First, our study is a single-center retrospective study based on electronic medical records. Thus, inevitable selection bias may be present. Second, due to the retrospective nature, potential bias and inaccuracy may inevitably exist in data collection, and measurement bias may be introduced into laboratory results. Third, RPR is extracted only at the time of admission, and the prognostic effects of dynamic changes in RDW, platelet, and RPR values have not been investigated. Fourth, relevant factors that may affect the levels of RDW, platelet, and RPR; like iron deficiency anemia, iron metabolism, erythropoietin use, and vitamin B12 shortfall, are not evaluated. Therefore, the predictive performance of RPR needs to be further verified by multicenter and prospective research.

## 5. Conclusion

Elevated RPR levels are significantly associated with increased 28-day mortality in patients with sepsis. Therefore, RPR can be used as a reliable and effective predictor of prognosis in patients with sepsis. However, more large prospective multicenter studies are needed to validate the results.

## Figures and Tables

**Table 1:** Comparison of baseline characteristics between 28-day survivors and nonsurvivors.

Variables	All patients (*n* = 3367)	Survivors (*n* = 2407)	Nonsurvivors (*n* = 960)	*P* value
Age (years)	66 [55, 77]	65 [54, 77]	69 [58,79]	< 0.001
Marital, *n* (%)				0.030
Married	1668 (49.5%)	1164 (48.4%)	504 (52.5%)	
Not married	1699 (50.5%)	1243 (51.6%)	456 (47.5%)	
Gender, *n* (%)				0.099
Female	1450 (43.1%)	1058 (44.0%)	392 (40.8%)	
Male	1917 (56.9%)	1349 (56.0%)	568 (59.2%)	
Ethnicity, *n* (%)				0.001
White	2449 (72.7%)	1756 (73.0%)	693 (72.2%)	
Black	352 (10.5%)	274 (11.4%)	78 (8.1%)	
Other	566 (16.8%)	377 (15.7%)	189 (19.7%)	
BMI (kg/m^2^)	27 [23, 32]	27 [24, 33]	27 [23, 32]	0.056
Temperature (°C)	37 [36, 37]	37 [36, 37]	37 [36, 37]	< 0.001
SpO_2_ (%)	97 [96, 99]	97 [96, 99]	97 [96, 99]	< 0.001
Renal replacement therapy, *n* (%)				0.001
No	3074 (91.3%)	2222 (92.3%)	852 (88.8%)	
Yes	293 (8.7%)	185 (7.7%)	108 (11.3%)	
Mechanical ventilation, *n* (%)				< 0.001
No	1586 (47.1%)	1223 (50.8%)	363 (37.8%)	
Yes	1781 (52.9%)	1184 (49.2%)	597 (62.2%)	
Vasopressor use, *n* (%)				< 0.001
No	997 (29.6%)	814 (33.8%)	183 (19.1%)	
Yes	2370 (70.4%)	1593 (66.2%)	777 (80.9%)	
Albumin (g/dL)	3 [2, 3]	3 [2,3]	3 [2, 3]	< 0.001
Bicarbonate (mEq/L)	22 [19, 25]	22 [19, 25]	21 [18, 25]	< 0.001
Anion gap (mEq/L)	15 [13,18]	15 [13, 17]	16 [14,19]	< 0.001
Creatinine (mg/dL)	1 [1, 2]	1 [1, 2]	2 [1, 3]	< 0.001
Bilirubin (mg/dL)	1 [0, 2]	1 [0, 2]	1 [1, 3]	< 0.001
Chloride (mEq/L)	105 [101, 109]	106 [102, 110]	104 [100, 109]	< 0.001
Glucose (mg/dL)	134 [110, 169]	134 [110, 168]	135 [110, 171]	0.772
Hematocrit (%)	31 [28, 35]	31 [28, 35]	30 [27, 34]	0.048
Hemoglobin (g/dL)	10 [9, 12]	10 [9, 12]	10 [9, 11]	0.012
Sodium (mEq/L)	138 [135, 141]	139 [135, 141]	138 [134, 141]	0.006
Potassium (mEq/L)	4 [4, 5]	4 [4, 5]	4 [4, 5]	< 0.001
BUN (mg/dL)	29 [18, 49]	27 [17, 45]	37 [23, 58]	< 0.001
WBC (10^9^/L)	13 [9, 19]	13 [9, 19]	13 [8, 19]	0.281
Lactate (mmol/L)	2 [1, 3]	2 [1, 3]	2 [2, 4]	< 0.001
PT (seconds)	16 [14, 19]	15 [14, 18]	16 [14, 21]	< 0.001
INR (seconds)	1 [1, 2]	1 [1, 2]	2 [1, 2]	< 0.001
Lymphocyte (%)	8 [4, 14]	9 [5, 15]	7 [3, 12]	< 0.001
Neutrophil (%)	82 [73, 89]	81 [72, 88]	83 [73, 90]	0.004
RPR	1 [1, 1]	1 [1, 1]	1 [1, 1]	< 0.001
Congestive heart failure, *n* (%)				0.001
No	2118 (62.9%)	1558 (64.7%)	560 (58.3%)	
Yes	1249 (37.1%)	849 (35.3%)	400 (41.7%)	
Hypertension, *n* (%)				0.550
No	1621 (48.1%)	1151 (47.8%)	470 (49.0%)	
Yes	1746 (51.9%)	1256 (52.2%)	490 (51.0%)	
Diabetes, *n* (%)				0.833
No	2225 (66.1%)	1588 (66.0%)	637 (66.4%)	
Yes	1142 (33.9%)	819 (34.0%)	323 (33.6%)	
SOFA	7 [4, 10]	8 [6, 11]	6 [4, 9]	< 0.001
SAPS II	44 [34, 54]	51 [42, 61]	41 [32, 51]	< 0.001

BMI: body mass index; SpO_2_: oxygen saturation; BUN: blood urea nitrogen; WBC: white blood cell; PT: prothrombin time; INR: international normalized ratio; RPR: red cell distribution width to platelet ratio; SOFA: sequential organ failure assessment; SAPS II: simplified acute physiology score II.

**Table 2:** Performance of red cell distribution width to platelet ratio for predicting 28-day mortality in patients with sepsis.

Variable	Cut-off	AUC (95% CI)	Sensitivity	Specificity
RPR	0.109	0.728 (0.709-0.747)	0.588	0.777
RDW	16.8	0.656 (0.636-0.677)	0.524	0.703
Platelet	0.006	0.628 (0.606-0.649)	0.513	0.700
SOFA	7.0	0.605 (0.584-0.626)	0.536	0.615
SAPS II	49.0	0.610 (0.589-0.631)	0.503	0.679

AUC: area under the curve; CI: confidence interval; RPR: red cell distribution width to platelet ratio; RDW: red cell distribution width; SOFA: sequential organ failure assessment; SAPS II: simplified acute physiology score II.

**Table 3:** Risk of 28-day mortality in patients with sepsis according to red cell distribution width to platelet ratio.

Model	Continuous variables		Categorical variable (≥ 0.109 vs. < 0.109)	
	HR (95% CI)	*P* value	HR (95% CI)	*P* value
Crude model	2.897 (2.216-3.788)	< 0.001	1.607 (1.415-1.825)	< 0.001
Model 1 ^a^	3.724 (2.846-4.872)	< 0.001	1.668 (1.468-1.895)	< 0.001
Model 2 ^b^	3.724 (2.846-4.872)	< 0.001	1.699 (1.493-1.932)	< 0.001
Model 3 ^c^	3.762 (2.761-5.126)	< 0.001	1.322 (1.149-1.522)	< 0.001
Model 4 ^d^	3.404 (2.483-4.666)	< 0.001	1.303 (1.132-1.500)	< 0.001
Model 5 ^e^	2.826 (2.025-3.944)	< 0.001	1.210 (1.045-1.400)	0.011

HR: hazard ratio; CI: confidence interval; ^a^ Model 1 was adjusted for demographic information, including age, gender, marital status, and ethnicity; ^b^ Model 2 also adjusted for complications including congestive heart failure, hypertension, and diabetes; ^c^ Model 3 additionally adjusted laboratory tests, including albumin, bicarbonate, anion gap, creatinine, bilirubin, chloride, glucose, hematocrit, hemoglobin, sodium, potassium, blood urea nitrogen, white blood cell, lactate, prothrombin time, international normalized ratio, lymphocyte, and neutrophil; ^d^ Model 4 made additional adjustments to clinical treatment, including renal replacement therapy, mechanical ventilation, and vasopressor use; ^e^ Model 5 additionally adjusted the scoring system, including sequential organ failure assessment and simplified acute physiology score II.

## Data Availability

This study was carried out using a free and open clinical database, namely, the Medical Information Mart for Intensive Care III (MIMIC-III).
